# Models that combine transcriptomic with spatial protein information exceed the predictive value for either single modality

**DOI:** 10.1038/s41698-021-00184-1

**Published:** 2021-05-28

**Authors:** Ioannis A. Vathiotis, Zhi Yang, Jason Reeves, Maria Toki, Thazin Nwe Aung, Pok Fai Wong, Harriet Kluger, Konstantinos N. Syrigos, Sarah Warren, David L. Rimm

**Affiliations:** 1 Department of Pathology, Yale School of Medicine, New Haven, CT USA; 2 Yale Cancer Center, Yale School of Medicine, New Haven, CT USA; 3 NanoString Technologies, Seattle, WA USA; 4 Section of Medical Oncology, Department of Internal Medicine, Yale School of Medicine, New Haven, CT USA; 5 Department of Medicine, National and Kapodistrian University of Athens School of Medicine, Athens, Greece

**Keywords:** Predictive markers, Immunohistochemistry

## Abstract

Immunotherapy has reshaped the field of cancer therapeutics but the population that benefits are small in many tumor types, warranting a companion diagnostic test. While immunohistochemistry (IHC) for programmed death-ligand 1 (PD-L1) or mismatch repair (MMR) and polymerase chain reaction (PCR) for microsatellite instability (MSI) are the only approved companion diagnostics others are under consideration. An optimal companion diagnostic test might combine the spatial information of IHC with the quantitative information from RNA expression profiling. Here, we show proof of concept for combination of spatially resolved protein information acquired by the NanoString GeoMx® Digital Spatial Profiler (DSP) with transcriptomic information from bulk mRNA gene expression acquired using NanoString nCounter® PanCancer IO 360™ panel on the same cohort of immunotherapy treated melanoma patients to create predictive models associated with clinical outcomes. We show that the combination of mRNA and spatially defined protein information can predict clinical outcomes more accurately (AUC 0.97) than either of these factors alone.

Combination immunotherapy targeting both cytotoxic T-lymphocyte associated protein 4 (CTLA-4) and programmed cell death 1 (PD-1) immune checkpoints has resulted in a median progression-free survival (PFS) of 11.5 months and 5-year survival rates of up to 52% in previously untreated patients with advanced melanoma.^1–3^ However, objective response to immune checkpoint inhibitors (ICI) is limited to 40% and evidence of clinical activity is present in up to 65% of patients.^4^

A number of methods have been tested for their predictive value for ICI therapy.^5^ PD-L1 expression by IHC on formalin-fixed, paraffin-embedded (FFPE) tissue has demonstrated limited predictive ability in patients with metastatic melanoma. PD-L1 IHC maintains suboptimal accuracy and reproducibility and offers limited information about the tumor microenvironment (TME).^6–8^ High tumor mutational burden (TMB) has also been correlated with response to ICI. However, TMB provides indirect and equivocal information about the immune response and has not been standardized yet.^9–11^ Recent studies have focused on generating gene expression profiles (GEP) to address all different cell types and phenotypes that comprise the complex TME and describe the crosstalk among different immune-regulatory pathways.^12–14^ GEPs have indeed proved more accurate in predicting response to ICI, however, transcriptomic assays lack spatial information that may provide context about the source of the transcript within the tumor microenvironment.^15^

To assess the relative power of both spatially informed protein combined with GEPs, we utilized a cohort of 59 retrospectively collected melanoma patients that received treatment with anti-PD-1 (34/59; nivolumab, pembrolizumab) or combination (25/59; ipilimumab plus nivolumab) immunotherapy in the metastatic setting at Yale Cancer Center (Supplementary Table 1).^16^ Unsupervised hierarchical clustering on the 770 mRNA and 132 protein variables (44 DSP targets, measured in three different compartments) revealed that DSP data mainly clustered away from bulk mRNA gene expression data, suggesting that RNA and protein bear discrete, mostly nonoverlapping pieces of biological information (Fig. 1a). Next, we compared normalized bulk mRNA counts to normalized protein counts in three different compartments (the melanocyte [s100/HMB45] compartment, the leukocyte [CD45] compartment and the macrophage [CD68] compartment) and the sum of all three compartments. We saw that mRNAs and protein products were best correlated in the melanocyte compartment, possibly reflecting both the abundance of tumor tissue after FFPE sample macrodissection as well as its transcriptional overactivity driving gene expression. Most protein derivatives exhibited a positive correlation with their corresponding mRNAs. We also found a particular set of proteins that showed weak correlation or anti-correlation with their mRNAs (*CD276, MLH1, MYC, BCL2, MSH2, MKI67, PMS2, CTNNB1, and STAT3*) (Fig. 1b).

**Fig. 1:**
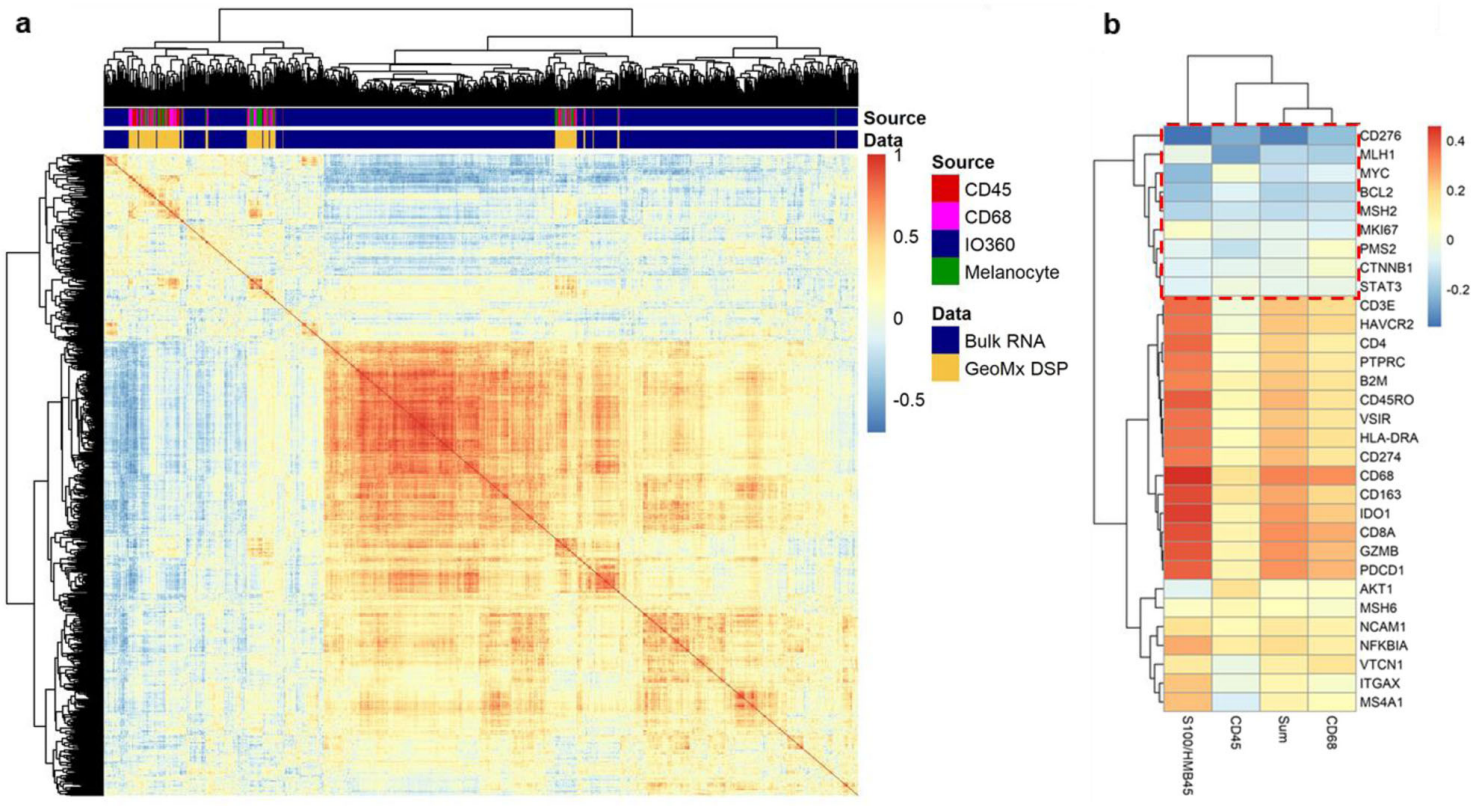
Correlation between mRNA and protein. **a** Unsupervised hierarchical cluster analysis for 770 mRNA targets acquired using NanoString nCounter® PanCancer IO 360™ panel and 44 protein targets acquired in three different compartments (s100/HMB45, CD45, and CD68) using NanoString GeoMx® Digital Spatial Profiler (DSP). DSP data generally cluster separately from bulk RNA profiling data. **b** Heatmap showing Spearman ranked correlation coefficient between bulk RNA and corresponding proteins quantified in three different compartments and the sum of all three compartments. A subset of protein targets shows weak correlation or anti-correlation to their precursor mRNAs (red dashed box).

Although currently challenging to assess on a single platform, combined modality (mRNA and protein) models may provide more detailed and comprehensive biological information, incorporating data related to immune regulation and other aspects of the tumor–stroma interaction and thus, prove superior to the existing models in predicting response to ICI. To explore this hypothesis, we extracted 527 variables that were modestly associated with the best overall response (BOR) (*p* < 0.10) by unadjusted univariate analysis (Fig. 2a). After removing moderately correlated predictors (*R*^2^ > 0.70), we used Elastic Net Regularization for feature selection and optimization for inclusion in different predictive models. We generated three models: a bulk mRNA gene expression model (*n* = 770 variables; PD/SD vs PR/CR, -0.23; *p* < 0.0001), a DSP model (*n* = 117 variables; PD/SD vs PR/CR, -0.04; *p* = 0.002) and a combined modality model (*n* = 44 variables, including 10 protein and 34 mRNA; PD/SD vs PR/CR, -0.68; *p* < 0.0001) (Fig. 2b). All proteins included in the DSP model were quantified in either the s100/HMB45 or the CD68 compartment. Although both PD-L1 mRNA and protein in the CD68 compartment were significantly associated with BOR, neither was selected in the combined modality model.

**Fig. 2:**
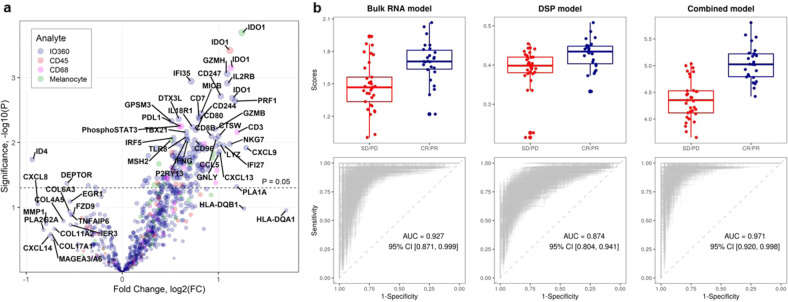
Combination of mRNA and protein improves best overall response (BOR) classification. **a** Identification of significant predictors for predictive model generation. Volcano plot showing mRNA and protein variables that are statistically significant for the prediction of BOR by unadjusted univariate analysis (*p* < 0.10, *n* = 527 variables, *p* < 0.05, *n* = 228 variables). **b** Combined modality model is superior to RNA-only or protein-only models in terms of BOR classification. Box and whisker plots and receiver operator characteristic (ROC) curves comparing a bulk RNA-only model (*n* = 770 variables; Area under the curve [AUC], 0.93; 95% confidence intervals [CI], 0.87–1.00; sensitivity, 0.93; specificity, 0.87; positive predictive value [PPV], 0.85; negative predictive value [NPV], 0.94) with a DSP-only model (*n* = 117 variables; AUC, 0.87; 95% CI, 0.80–0.94; sensitivity, 0.79; specificity, 0.88; PPV, 0.84; NPV, 0.84) and a combined bulk RNA and DSP model (*n* = 44 variables; AUC, 0.97; 95 CI, 0.92-1.00; sensitivity, 0.96; specificity, 0.93; PPV, 0.91; NPV, 0.96); feature selection occurs through Elastic Net Regularization after removal of moderately correlated predictors (*R*^2^ > 0.70). On each boxplot, the central line indicates the median and edges indicate the interquartile range. The upper whisker extends from the 75th percentile to the largest value at most the 1.5x interquartile and the lower whisker extends from the 25th percentile to the smallest value at most the x1.5 interquartile.

We observed improvement in the classification of BOR for the 44-variable combined modality model (Area under the curve [AUC], 0.97; 95 confidence intervals [CI], 0.92 to 1.00; sensitivity, 0.96; specificity, 0.93; positive predictive value [PPV], 0.91; negative predictive value [NPV], 0.96). This exceeded the AUC for both the 770-variable transcriptomic model (AUC, 0.93; 95% CI, 0.87–1.00; sensitivity, 0.93; specificity, 0.87; PPV, 0.85; NPV, 0.94) and the 117-variable DSP model (AUC, 0.87; 95% CI, 0.80–0.94; sensitivity, 0.79; specificity, 0.88; PPV, 0.84; NPV, 0.84). Model improvement was more prominent over DSP rather than bulk mRNA, possibly because of the increased number (~ 5-fold) of features derived from the RNA dataset that were introduced in the analysis. The features, or variables, while fractionally increasing the AUC, can make the application of the model impractical or non-reproducible.

Toward the goal of generating a clinical test, we extended the analysis of the data to find the minimal subset of variables that need to be included in the model without causing substantial decline in model performance, representing the optimal trade-off between efficacy and simplicity. To accomplish this, we ranked the top ten sets of variables that demonstrated the highest predictive ability, based on AUC, for any given number of variables included in the model (K). Then, we constructed new sets that were composed of the most frequently appearing variables for each K value. Finally, we calculated AUC, sensitivity, specificity, PPV, and NPV to compare these sets for K values between 4 and 13 (Fig. 3a and Supplementary Fig. 1a–d). The first peak in all five curves was observed when the number of variables was equal to eight (K = 8). Hence, we selected the 8-variable (*CCNO*, *ID4*, *IER3*, *IL2RB*, *MGMT*, *NRDE2*, *TNFAIP6,* and MSH2 in s100/HMB45) (Fig. 3b) hereafter referred to as the Yale Mixed Modality Model (YMMM) (Supplementary Table 2). We then tested the YMMM for the prediction of BOR to ICI in patients with advanced melanoma (AUC, 0.88; 95% CI, 0.78–0.95; sensitivity, 0.85; specificity, 0.83; PPV, 0.79; NPV, 0.88) (Fig. 4a, b). It was apparent that YMMM incorporated three distinct components; a component pertaining to cell cycle regulation and oncogenesis (*CCNO*, *ID4,* and *IER3*), a component related to unrepaired DNA damage, accumulation of mutations, and microsatellite instability (*MGMT*, *NRDE2,* and MSH2 in S100/HMB45) and a component directly linked with the immune response towards the primary tumor (*IL2RB* and *TNFAIP6*).^17–21^

**Fig. 3:**
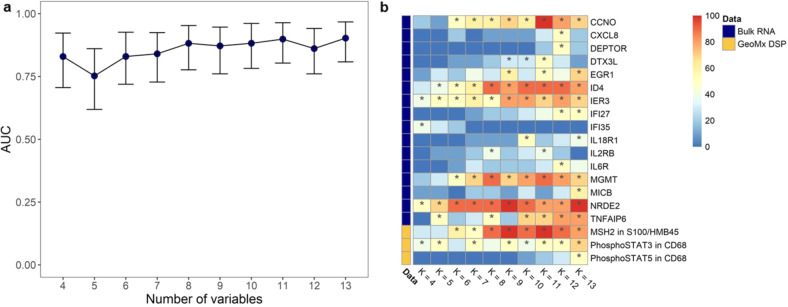
Generation of Yale Mixed Modality Model (YMMM) for the prediction of best overall response to immunotherapy in patients with advanced melanoma. **a** Identification of the optimal number of predictors for final model inclusion. Area under the curve (AUC) and 95% confidence intervals based on the number of predictors included in the model; AUC curve peaks when 8 predictors are included in the model. **b** Heatmap showing the most frequently appearing predictors for any given number of variables included in the model (K); calculations are based on the top ten highest AUC models for different K values. For *K* = 8, selected predictors are *CCNO*, *ID4*, *IER3*, *IL2RB*, *MGMT*, *NRDE2*, *TNFAIP6,* and MSH2 in s100/HMB45.

**Fig. 4:**
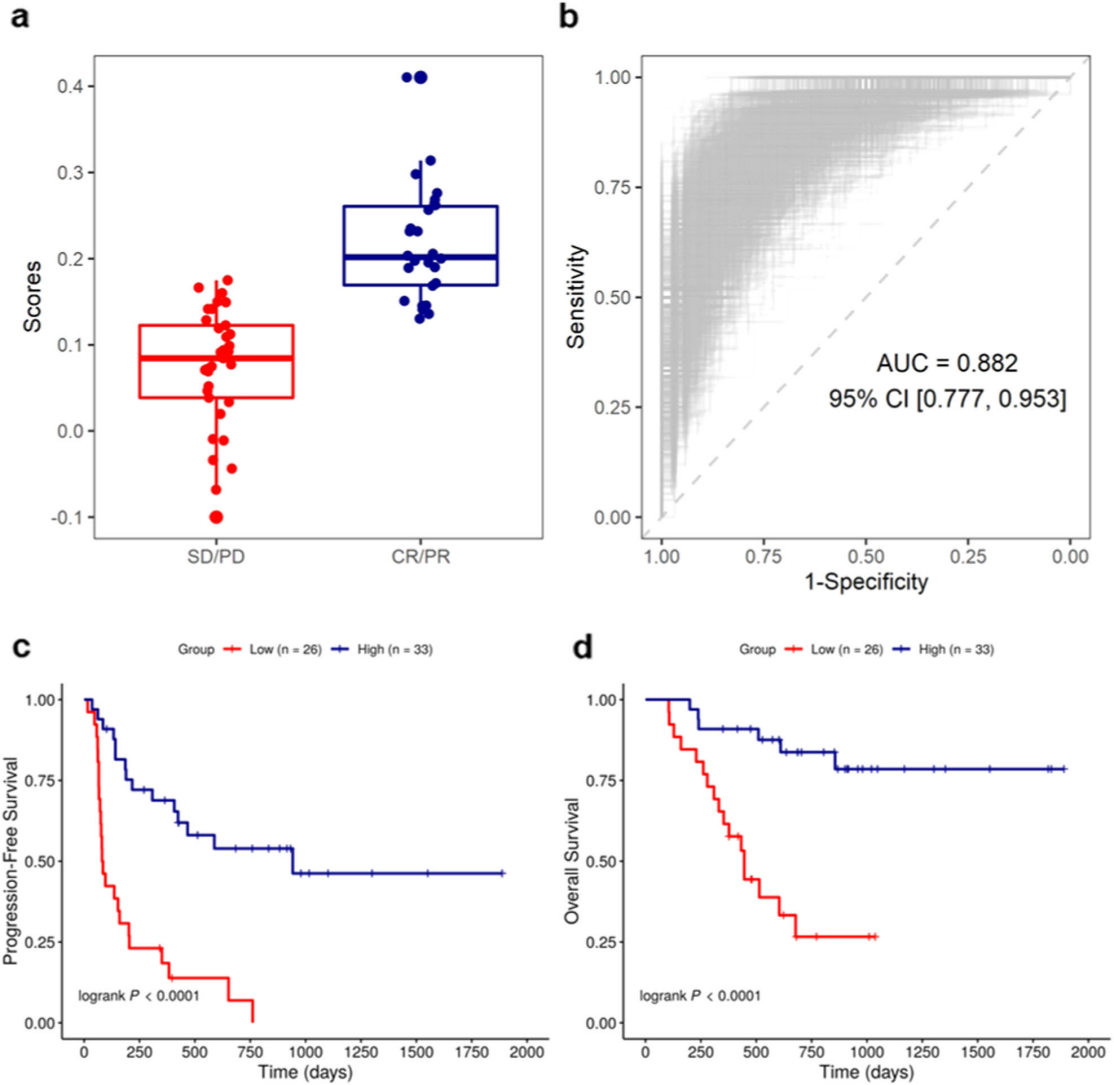
Yale Combined Modality Model (YMMM) predicts response to immunotherapy in patients with advanced melanoma. **a**, **b** Predictive value of YMMM for best overall response (BOR). Box and whisker plot (**a**) and receiver operator characteristic (ROC) curve (**b**) for the prediction of BOR (AUC, 0.88; 95% CI, 0.78 to 0.95; sensitivity, 0.85; specificity, 0.83; PPV, 0.79; NPV, 0.88); on each boxplot, the central line indicates the median and edges indicate the interquartile range. The upper whisker extends from the 75th percentile to the largest value at most the 1.5x interquartile and the lower whisker extends from the 25th percentile to the smallest value at most the ×1.5 interquartile. **c** Predictive value of YMMM for progression-free survival (PFS). Kaplan–Meier curve showing that patients with high YMMM score have significantly prolonged PFS in comparison with patients with low YMMM score (HR, 0.20; 95% CI, 0.10-0.41; *p* < 0.0001). **d** Predictive value of YMMM for overall survival (OS). Kaplan–Meier curve showing that patients with high YMMM score have significantly prolonged OS in comparison with patients with low YMMM score (HR, 0.16; 95% CI, 0.06-0.43; *p* < 0.0001). Cutoff point for high and low-risk subgroup stratification was calculated based on Youden’s index.

YMMM performance for the prediction of progression-free survival (PFS) and overall survival (OS) needs to be considered in the context for which it will be used. For this analysis, we calculated the optimal, based on the Youden’s index, cutpoint for the prediction of BOR and used it to create high and low risk subgroups. Patients with high score according to YMMM performed significantly better in terms of both PFS (HR, 0.20; 95% CI, 0.10–0.41; log rank *p* < 0.0001) and OS (HR, 0.16; 95% CI, 0.06–0.43; log rank *p* < 0.0001) in comparison with patients with low YMMM score (Fig. 4c, d). Previous studies have demonstrated that conventional biomarkers carry suboptimal predictive ability for melanoma patients treated with ICI, as they are only able to illuminate one or limited aspects of the tumor-TME interaction and are designed to implement binary patient stratification (positive/negative), failing to incorporate the dynamic range of responses to this particular type of therapy.^22^ YMMM represents a multimodality approach that selects and encompasses essential information about multiple elements related to response to ICI. Furthermore, it functions as a continuous score, rather than a binary variable, enabling precise as well as dynamic benefit stratification to optimize clinical decision making.

But in practice, especially in the metastatic setting, many predictive assays do not use the optimal area under the curve since it is critical to provide patients the greatest opportunity to benefit by maximizing sensitivity. An example of this is ERBB2 in breast cancer. The current assay combination of IHC, then FISH has high sensitivity (as high as 95%) but relatively low specificity^23,24^ for predictive response to HER2 targeting therapy. In fact, even in the adjuvant setting where 65–70% of patients showed long-term survival with placebo,^25^ the same assay is used in effort to leave no patient behind, although many patients will not benefit from the drug (low specificity). Similarly, as we build the YMMM assay with a limited, accessible and highly reproducible biomarker set, we need to design the assay for high sensitivity, even at the expense of specificity. Our model suggests that for prediction of response with 95% sensitivity the assay would have a specificity between 0.63 and 0.94.

In summary, this is a proof of concept study and further analyses are required to construct and validate the YMMM. As such, a limitation of this work is the absence of validation using cohorts in the literature since no previous cohort has collected both spatial protein and transcriptomic information. Another limitation is the relatively small size of the cohort and the fact that it is comprised of patients that received either single-agent or combination immunotherapy. Future studies are planned to validate the 8-variable YMMM, including retrospective collections of patients with melanoma, as well as other tumor types, treated with ICI and ultimately, prospective clinical trials. In addition, YMMM or similar models should be correlated with other predictive assays, including PD-L1 IHC score and TMB. Finally, in an era where immunotherapy indications are relentlessly expanding, YMMM or similarly constructed mixed modality models could be used to develop predictors for single agents or therapeutic combinations that may have distinct, compartment-specific mechanisms of action.

## Methods

### Tissue microarray and patient cohorts

Tissue specimens were prepared in a tissue microarray (TMA) format as described previously.^26^ After review by a board-certified pathologist, representative 0.6 mm cores from areas with high tumor content were obtained from FFPE specimens and arrayed in a recipient block. FFPE normal tissue was used as a control. All specimens were collected from the Yale Pathology archives. The study cohort (YTMA376) is a retrospective collection of 59 pretreatment melanoma tumor specimens resected between 2011 and 2016. Uveal melanoma was excluded. The corresponding patients were treated with anti-PD-1 (nivolumab, pembrolizumab) or combination (ipilimumab plus nivolumab) immunotherapy in the metastatic setting at Yale Cancer Center. Clinicopathological data were collected from clinical records and pathology reports; the data cut-off date was September 1, 2017.^27^ Response Evaluation Criteria in Solid Tumors (RECIST) 1.1 were used to determine best overall response (BOR) as complete response (CR), partial response (PR), stable disease (SD), or progressive disease (PD), and objective response rate (ORR; CR/PR), durable clinical benefit rate (DCBR; CR/PR/SD ≥ 6 months), disease control rate (DCR; CR/PR/SD).^28^ All patients provided written informed consent or waiver of consent. The study was approved by the Yale Human Investigation Committee protocol #9505008219 and conducted in accordance with the Declaration of Helsinki.

### mRNA gene expression

For the gene expression analysis, pretreatment FFPE whole tissue sections from the 59 melanoma patients included in YTMA 376 were employed. Two slides from each patient were macrodissected and RNA was extracted. The mRNA transcripts were hybridized to 4-color, 6-spot optical barcodes, exclusive for each of the targets included in the 770-plex PanCancer IO360 panel. Barcodes were then measured by a fluorescence microscope on the nCounter platform. Finally, RNA counts were normalized for technical efficiency by the geometric mean of internal control probes, and then, to account for sample-specific RNA content, against the geometric mean housekeeping genes present on the panel. For analysis, normalized counts were log_2_ transformed.

### Digital spatial profiling

The NanoString DSP is a novel platform that allows spatially-resolved, high-plex quantitative measurement of target proteins on a single FFPE slide. In this study, TMA slides were incubated with cocktails of 44 unique, previously validated, oligonucleotide-conjugated antibodies (Extended Data Table 3). Each TMA spot was represented by a unique region of interest (ROI). We hypothesized that immune markers, including immune checkpoints, have differential expression patterns among immune cell populations that comprise the tumor microenvironment and carry different predictive significance with respect to the cell type that they are expressed. So, on each ROI, different compartments, called areas of interest (AOI), were created based on fluorescent staining with antibodies targeting s100 with HMB45 for melanocytes, CD45 for tumor-infiltrating leukocytes, and CD68 for tumor-infiltrating macrophages (Extended Data Fig. 2). Oligos from each AOI were then released upon exposure to UV light. Photocleaved oligos were collected via microcapillary tube inspiration by sequential assignment of the CD68 + , CD45 + , and finally s100/HMB45 + AOI and transferred into a microwell plate with a spatial resolution of approximately 10 mm. Photocleaved oligos were then hybridized to 4-color, 6-spot optical barcodes producing uniquely labeled tags per AOI for each of the 44 antibodies included in the original mix. Digital counts from barcodes corresponding to protein probes were first normalized with internal positive and negative controls to account for system variation, and then normalized to the area of their compartment.

### Statistical analysis and predictive model generation

After excluding five controls from the analysis including Histone H3, Mouse IgG1, Mouse IgG2a, Rabbit IgG, and S6, a total of 887 targets (770 + (44 – 5) × 3) remained to build an elastic net regularized regression model for predicting BOR. A more predictive subset of variables (*n* = 527) was formed with *p-*value less than 0.10 in univariate logistic regression models. To minimize the multicollinearity issue among 527 predictors, an iterative pruning procedure was performed by ranking predictors in descending order of its univariate R-squared in predicting BOR and only keeping those with the highest AUC by removing other moderately correlated predictors (correlation coefficient > 0.7). Therefore, only 72 predictors with pair-wise correlation coefficients less than 0.7 remained to enter the next phase of modeling training to tune two important parameters in regularization models, the elastic net mixing parameter α and the regularization parameter λ. Melanoma tumor specimens were split into 80% training set and 20% testing set stratified by BOR. Models were built on the training set in which α and λ were tuned simultaneously in four-fold cross-validation.^29^ AUC values were used to evaluate model performance and to select the optimal parameters. The process was performed by looping across levels of α ranging from 0 to 1 in steps of 0.05 in which λ was selected at the highest value of AUC for a given α. To stabilize the tuning process for the parameter determination, the previous looping step was performed for 40 replicates to obtain the maximized averaged AUC values for the best combination of parameters (α = 0.15, λ = 0.642). To further reduce overfitting on training a small dataset, instead of fitting the entire data with the tuned parameters, an optimal subset of predictors was constructed by those most frequently selected predictors, with non-zero coefficients, from fitting the elastic net models on bootstrapped data over 1000 replicates, which returned a model size of 59 at its median value. Then, α = 0.15, λ = 0.642 were applied to the data consisting of the top 59 most frequently selected predictors in which 44 of 59 returned non-zero coefficients. Results of utilizing both proteins and bulk mRNA were compared to two other scenarios where either only proteins or bulk mRNA were used in model building. To further assess the predictive performance of different combinations within these 44 targets, the top ten highest AUC with corresponding targets were recorded to determine the most predictive subset over a range of the number of desirable predictors, K, from 4 to 13. It is noted that the following results are based on the coefficient derived from the final model without refitting any new models. When K is greater than five, 2,000,000 unique combinations were created using the Monte Carlo method instead of an exhaustive search of all combinations of all predictors. Among all possible combinations of a given size of K, predictors were ranked by its frequency based on the results from the top ten highest AUC value in which K number of predictors were selected. To calculate the 95% confidence intervals of AUC, sensitivity, specificity, positive predictive value, and negative predictive value the smoothed bootstrap from the kernel boot package was applied to draw samples with replacement from the empirical distribution for 1,000 times which estimates the uncertainty of each measurement.^30^ The best subset of eight predictors (*CCNO*, *ID4*, *IER3*, *IL2RB*, *MGMT*, *NRDE2*, *TNFAIP6,* and MSH2 in s100/HMB45) was selected which had the largest improvement on the AUC value. The variable importance was calculated based on the decrease in AUC after 1,000 replicates of permutation in each predictor.^31^ Signature scores of these eight predictors, the sum of the product of expression level and coefficients, were used to estimate the AUC value and 95% CI in predicting BOR. Kaplan-Meier analyses were performed on overall survival and progression-free survival data between high-score groups and low-score groups, which the cutoff of scores was determined at the highest Youden’s index in predicting BOR.^32^ The entire analysis was performed using R 3.6.3.

### Reporting summary

Further information on research design is available in the Nature Research Reporting Summary linked to this article.

## Supplementary information

Supplementary Information

Reporting Summary

## Data Availability

The data generated and analyzed during this study are described in the following data record: 10.6084/m9.figshare.14345894.^33^ The data are housed in Yale AQUAmine in the files ‘376_2_1_Nanostring_IO360_panel_IxV.txt’, ‘376_1_3_Nanostring_2nd_run_immune_panel_mxt.txt’ and ‘376_3_2_Nanostring_immune_panel_mxt.txt’. These files are not publicly available as they contain information that could compromise research participant privacy. However, the data can be made available upon reasonable request to the corresponding author Dr David L Rimm.

## Code availability

The data were processed and analyzed using R version 3.6.3 which is tested on both Linux and Windows systems. The R packages and versions used are kernelboot(0.1.7), caret(6.0-86), lattice(0.20-41), OptimalCutpoints(1.1-4), glmnetUtils(1.1.5), patchwork(1.0.0), survminer(0.4.6), ggpubr(0.2.5), magrittr(1.5), pROC(1.16.2), DT(0.13), glmnet(3.0-2), Matrix(1.2-18), survival(3.1-8), pheatmap(1.0.12), ggrepel(0.8.2), ggplot2(3.3.0), readxl(1.3.1), and rsq(1.1). The datasets generated and/or analyzed during the current study are available in the data folder of the GitHub repository, https://github.com/Nanostring-Biostats/TSCOL_0137-Vathiotis_Rimm.
