# Supplementary material for: Models that combine transcriptomic with spatial protein information exceed the predictive value for either single modality
Source: NPJ Precis Oncol. 2021 May 28;5:45. doi: 10.1038/s41698-021-00184-1 (PMC8163775; doi:10.1038/s41698-021-00184-1)
Supplement: Supplementary file 1 — Supplementary Information [file 41698_2021_184_MOESM1_ESM.pdf]

**Characteristic**	**N (%)**
Overall	59 (100)
Age (years)	39 (66)
<70	20 (34)
≥70	
Sex	33 (56)
Male	26 (44)
Female	
Mutation status	18 (31)
BRAF	8 (14)
NRAS	1 (2)
CKIT	32 (54)
None	
Stage at diagnosis	10 (17)
I	11 (19)
II	22 (37)
III	12 (20)
IV	4 (7)
Not available	
Treatment	23 (39)
Pembrolizumab	11 (19)
Nivolumab	25 (42)
Ipilimumab plus nivolumab	
Best overall response	10 (17)
Complete response	16 (27)
Partial response	17 (29)
Stable disease	16 (27)
Progressive disease	
Durable clinical benefit	36 (61)
Yes	23 (39)
No	

Supplementary Table 1. Patient characteristics.

Variable	Coefficient
CCNO	0.0181943
ID4	-0.030076
IER3	-0.006943
MSH2 in s100/HMB45	0.0547323
MGMT	0.0386636
NRDE2	-0.06098
TNFAIP6	-0.009739
IL2RB	0.0320801

Supplementary Table 2. Variables and corresponding coefficients that comprise the YMMM.

#	Target	CAB
1	Beta-Catenin	963
2	PMS2	934
3	CD8	1022
4	B7-H3	959
5	CD4	941
6	Phospho-p70S6K	336
7	Mouse IgG1	891
8	CD68	1023
9	Phospho-RPS6	767
10	Rabbit IgG	836
11	MLH1	937
12	CD34	1041
13	MSH2	919
14	S6	837
15	GZMB	1068
16	Ki-67	314
17	Beta-2-microglobulin	964
18	Histone H3	387
19	Akt	962
20	CD3	1019
21	MSH6	920
22	VISTA	940
23	PD1	1024
24	Phospho-STAT3	458
25	STAT3	957
26	CD56	1020
27	PD-L1	1039
28	CD45	1018
29	Tim3	1038
30	HLA-DR	1052
31	CD20	706
32	Pan-Cytokeratin	886
33	CD45RO	77
34	Bcl-2	960
35	B7-H4	1053
36	Ik-Ba	907
37	BIM	894
38	IDO1	1033
39	CD11c	1036
40	Mouse IgG2a	1258
41	BCL6	880
42	c-Myc	878
43	Phospho-STAT5	882
44	CD163	1031

Supplementary Table 3. DSP antibody panel. CAB is custom antibody ID, which uniquely identifies this reagent.

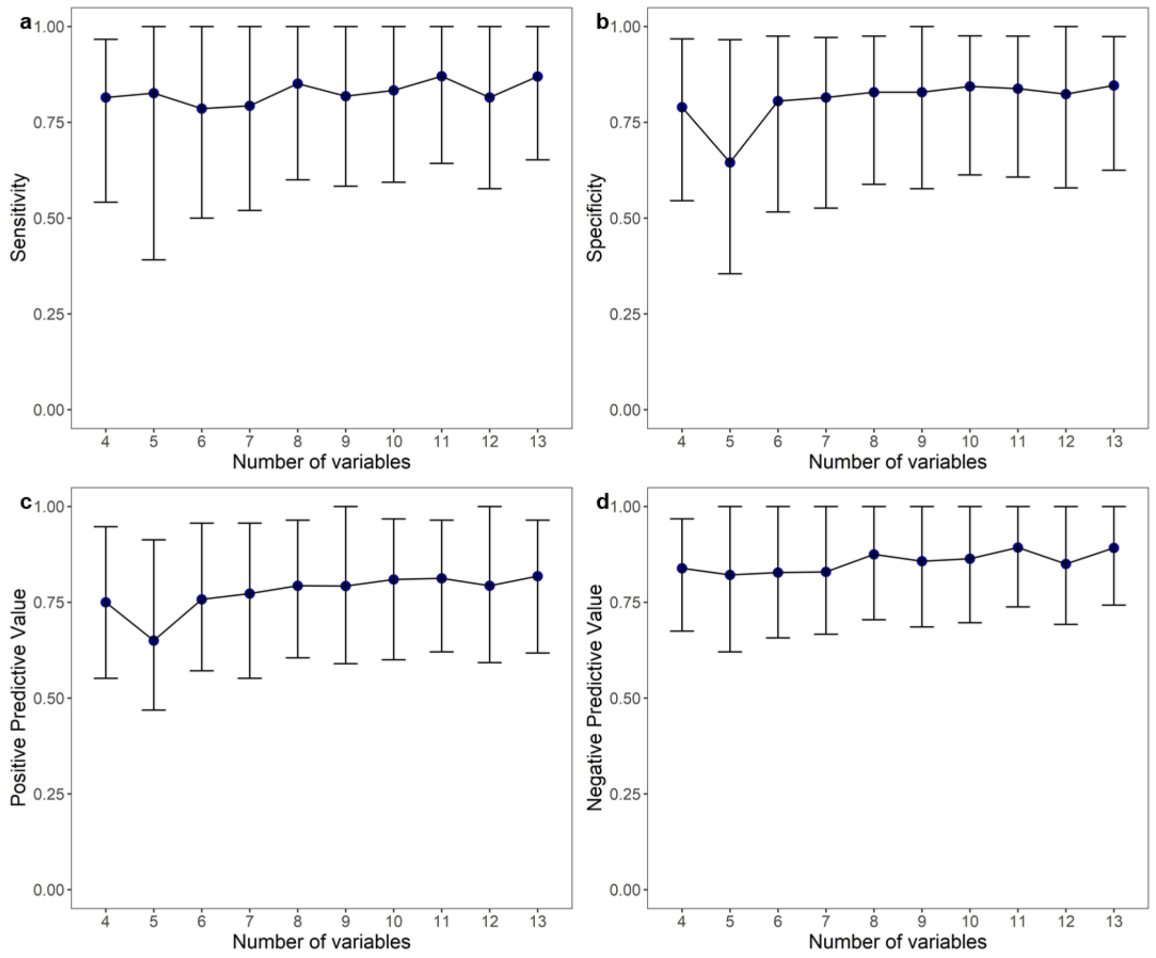

Supplementary Figure 1. Identification of the optimal number of predictors for final model inclusion. a-d, Sensitivity (**a**), specificity (**b**), positive predictive value (**c**) and negative predictive value (**d**) and 95% confidence intervals based on the number of predictors included in the model; all curves peak when 8 predictors are included in the model.

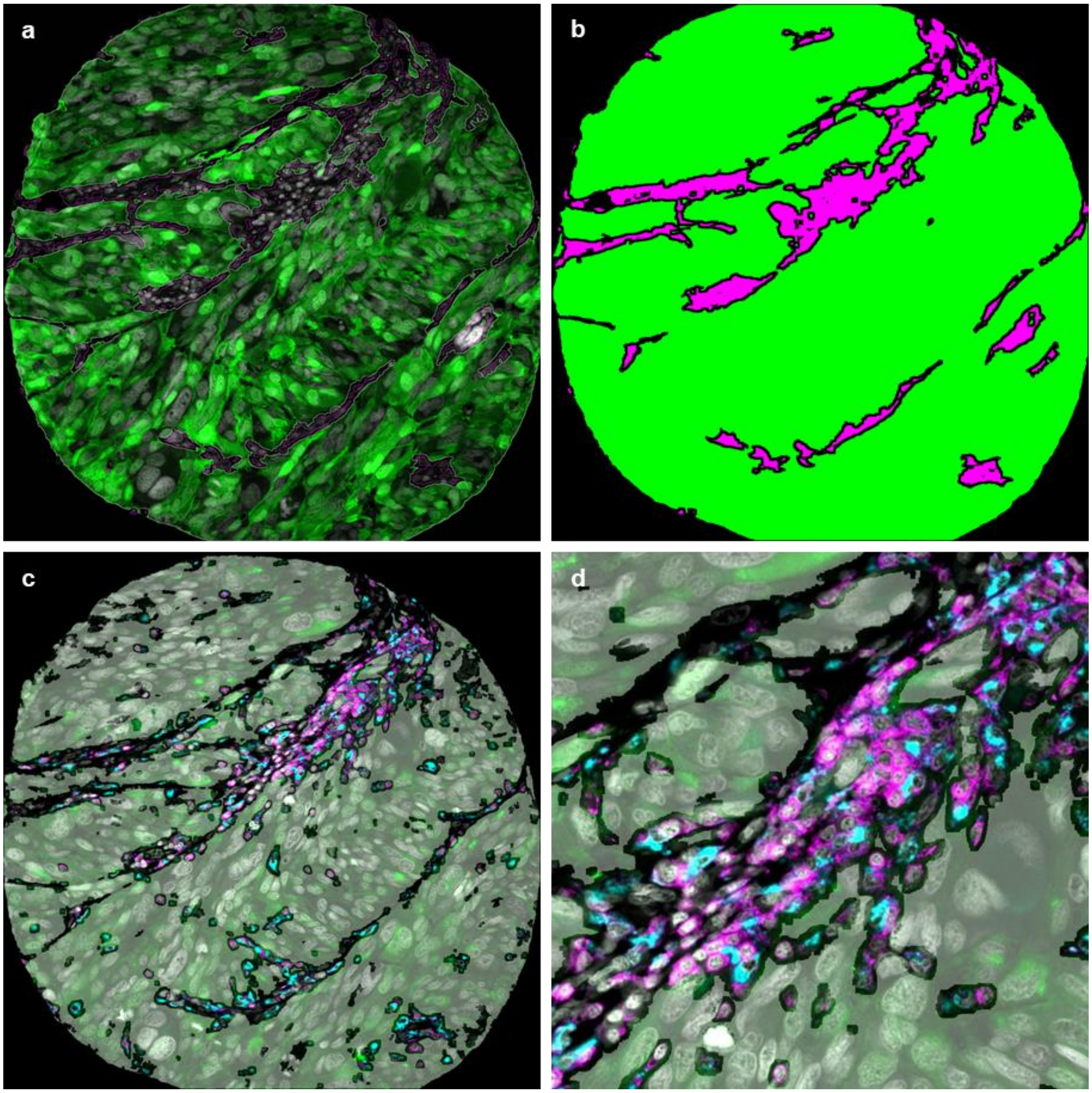

Supplementary Figure 2. Representative image of DSP compartment selection. a-b, Tumor compartment (green) is defined by s100/HMB45 positivity, while stromal compartment (purple) is defined by s100/HMB45 negativity. **c-d**, Stroma is further divided to macrophage compartment that is CD68-positive (cyan) and leukocyte compartment that is CD45+ (magenta).
