# Supplementary material for: Models that combine transcriptomic with spatial protein information exceed the predictive value for either single modality
Source: NPJ Precis Oncol. 2021 May 28;5:45. doi: 10.1038/s41698-021-00184-1 (PMC8163775; doi:10.1038/s41698-021-00184-1)
Supplement: Supplementary file 2 — Reporting Summary [file 41698_2021_184_MOESM2_ESM.pdf]

nature research

Corresponding author(s): David L. Rimm

Last updated by author(s): Dec 9, 2020

Reporting Summary

Nature Research wishes to improve the reproducibility of the work that we publish. This form provides structure for consistency and transparency in reporting. For further information on Nature Research policies, see our Editorial Policies and the Editorial Policy Checklist.

Statistics

For all statistical analyses, confirm that the following items are present in the figure legend, table legend, main text, or Methods section.

n/a	Confirmed	
☐	☒	The exact sample size (*n*) for each experimental group/condition, given as a discrete number and unit of measurement
☐	☒	A statement on whether measurements were taken from distinct samples or whether the same sample was measured repeatedly
☐	☒	The statistical test(s) used AND whether they are one- or two-sided<br>*Only common tests should be described solely by name; describe more complex techniques in the Methods section.*
☐	☒	A description of all covariates tested
☐	☒	A description of any assumptions or corrections, such as tests of normality and adjustment for multiple comparisons
☐	☒	A full description of the statistical parameters including central tendency (e.g. means) or other basic estimates (e.g. regression coefficient) AND variation (e.g. standard deviation) or associated estimates of uncertainty (e.g. confidence intervals)
☐	☒	For null hypothesis testing, the test statistic (e.g. *F*, *t*, *r*) with confidence intervals, effect sizes, degrees of freedom and *P* value noted<br>*Give P values as exact values whenever suitable.*
☒	☐	For Bayesian analysis, information on the choice of priors and Markov chain Monte Carlo settings
☒	☐	For hierarchical and complex designs, identification of the appropriate level for tests and full reporting of outcomes
☐	☒	Estimates of effect sizes (e.g. Cohen's *d*, Pearson's *r*), indicating how they were calculated

Our web collection on statistics for biologists contains articles on many of the points above.

Software and code

Policy information about availability of computer code

Data collection	*Provide a description of all commercial, open source and custom code used to collect the data in this study, specifying the version used OR state that no software was used.*
Data analysis	The data were processed and analyzed using R version 3.6.3 which are tested on both Linux and Windows systems. The R packages and version are listed including kernelboot(0.1.7), caret(6.0-86), lattice(0.20-41), OptimalCutpoints(1.1-4), glmnetUtils(1.1.5), patchwork(1.0.0), survminer(0.4.6), ggpubr(0.2.5), magrittr(1.5), pROC(1.16.2), DT(0.13), glmnet(3.0-2), Matrix(1.2-18), survival(3.1-8), pheatmap(1.0.12), ggrepel(0.8.2), ggplot2(3.3.0), readxl(1.3.1), and rsq(1.1).

For manuscripts utilizing custom algorithms or software that are central to the research but not yet described in published literature, software must be made available to editors and reviewers. We strongly encourage code deposition in a community repository (e.g. GitHub). See the Nature Research guidelines for submitting code & software for further information.

Data

Policy information about availability of data

All manuscripts must include a data availability statement. This statement should provide the following information, where applicable:

- Accession codes, unique identifiers, or web links for publicly available datasets
- A list of figures that have associated raw data
- A description of any restrictions on data availability

The datasets generated during and/or analysed during the current study are available in the data folder of the GitHub repository, https://github.com/Nanostring-Biostats/TSCOL_0137-Vathiotis_Rimm.

Field-specific reporting

Please select the one below that is the best fit for your research. If you are not sure, read the appropriate sections before making your selection.

☒ Life sciences ☐ Behavioural & social sciences ☐ Ecological, evolutionary & environmental sciences

For a reference copy of the document with all sections, see nature.com/documents/nr-reporting-summary-flat.pdf

Life sciences study design

All studies must disclose on these points even when the disclosure is negative.

Sample size	Retrospective collection of melanoma patients treated with immunotherapy at Yale Cancer Center up to September 1st, 2017.
Data exclusions	Patients with uveal melanoma were excluded.
Replication	All patient samples were acquired in 2-fold redundancy.
Randomization	No randomization due to retrospective character of the study.
Blinding	Blinding was not possible due to retrospective character of the study.

Reporting for specific materials, systems and methods

We require information from authors about some types of materials, experimental systems and methods used in many studies. Here, indicate whether each material, system or method listed is relevant to your study. If you are not sure if a list item applies to your research, read the appropriate section before selecting a response.

Materials & experimental systems

n/a	Involved in the study	
☐	☒	Antibodies
☒	☐	Eukaryotic cell lines
☒	☐	Palaeontology and archaeology
☒	☐	Animals and other organisms
☐	☒	Human research participants
☐	☒	Clinical data
☒	☐	Dual use research of concern

Methods

n/a	Involved in the study	
☒	☐	ChIP-seq
☒	☐	Flow cytometry
☒	☐	MRI-based neuroimaging

Antibodies

Antibodies used	NanoString GeoMX DSP antibody panel.
Validation	Previously validated by NanoString.

Human research participants

Policy information about studies involving human research participants

Population characteristics	See Extended Data Table 1| Patient characteristics.
Recruitment	Retrospective collection of melanoma patients treated with immunotherapy at Yale Cancer Center up to September 1st, 2017.
Ethics oversight	The study was approved by the Yale Human Investigation Committee protocol #9505008219 and conducted in accordance with the Declaration of Helsinki.

Note that full information on the approval of the study protocol must also be provided in the manuscript.

Clinical data

Policy information about clinical studies

All manuscripts should comply with the ICMJE guidelines for publication of clinical research and a completed CONSORT checklist must be included with all submissions.

Clinical trial registration	Not applicable- work done on a retrospective collection, not a clinical trial.
Study protocol	Not applicable- work done on a retrospective collection, not a clinical trial.
Data collection	Not applicable- work done on a retrospective collection, not a clinical trial.
Outcomes	Outcomes assessed include best overall response as complete response (CR), partial response (PR), stable disease (SD), or progressive disease (PD), and objective response rate (ORR; CR/PR), clinical benefit rate (CBR; CR/PR/SD ≥ 6 months), disease control rate (DCR; CR/PR/SD).
